# Efficient production and characterization of the novel and highly active antifungal protein AfpB from *Penicillium digitatum*

**DOI:** 10.1038/s41598-017-15277-w

**Published:** 2017-11-07

**Authors:** Sandra Garrigues, Mónica Gandía, Crina Popa, Attila Borics, Florentine Marx, María Coca, Jose F. Marcos, Paloma Manzanares

**Affiliations:** 1 Department of Biotechnology, Instituto de Agroquímica y Tecnología de Alimentos (IATA), Consejo Superior de Investigaciones Científicas (CSIC), Paterna, Valencia, Spain; 2 Centre for Research in Agricultural Genomics (CRAG), CSIC-IRTA-UAB-UB. Edifici CRAG, Bellaterra, Barcelona, Spain; 3 Institute of Biochemistry, Biological Research Centre of Hungarian Academy of Sciences, Szeged, Hungary; 4 Biocenter, Division of Molecular Biology, Medical University of Innsbruck, Innsbruck, Austria

## Abstract

Filamentous fungi encode distinct antifungal proteins (AFPs) that offer great potential to develop new antifungals. Fungi are considered immune to their own AFPs as occurs in *Penicillium chrysogenum*, the producer of the well-known PAF. The *Penicillium digitatum* genome encodes only one *afp* gene (*afpB*), and the corresponding protein (AfpB) belongs to the class B phylogenetic cluster. Previous attempts to detect AfpB were not successful. In this work, immunodetection confirmed the absence of AfpB accumulation in wild type and previous recombinant constitutive *P*. *digitatum* strains. Biotechnological production and secretion of AfpB were achieved in *P*. *digitatum* with the use of a *P*. *chrysogenum*-based expression cassette and in the yeast *Pichia pastoris* with the α-factor signal peptide. Both strategies allowed proper protein folding, efficient production and single-step purification of AfpB from culture supernatants. AfpB showed antifungal activity higher than the *P*. *chrysogenum* PAF against the majority of the fungi tested, especially against *Penicillium* species and including *P*. *digitatum*, which was highly sensitive to the self-AfpB. Spectroscopic data suggest that native folding is not required for activity. AfpB also showed notable ability to withstand protease and thermal degradation and no haemolytic activity, making AfpB a promising candidate for the control of pathogenic fungi.

## Introduction

Nowadays fungal infections have become a serious threat to human health and food security. Human fungal infections have severe consequences on the growing number of immune-compromised patients with high mortality rates. In addition, the control of plant diseases caused by phytopathogenic fungi represents a big challenge in agriculture. The emergence of antifungal resistant strains is in continuous growth, emphasizing the urgent need for the development of novel antifungal agents with properties and mechanisms of action different from existing ones^1^.

Antifungal proteins (AFPs) secreted by filamentous fungi have been considered promising candidates for the development of novel antifungal compounds and therapies. AFPs are small, highly stable, cationic, cysteine-rich proteins (CRPs) stabilized by up to four disulphide bridges^2^. They are usually secreted in high amounts by filamentous Ascomycetes, mainly from the genera *Penicillium* and *Aspergillus*, and show potent antifungal activity against non-self fungi at micromolar concentrations.

It is increasingly clear that fungi have a complex repertoire of AFPs that offers a great potential to obtain new antifungal agents. In a previous study, fungal genome sequences were searched to identify AFP-like sequences and conduct detailed phylogenetic studies^3^. Based on phylogenetic clustering but also on sequence alignment, cysteine pattern, intron position and Pfam domain identification, we proposed the classification of fungal AFPs into three classes: A, B and C^3^, expanding the two previously reported ones that divided the fungal AFPs into two clusters^4,5^. Recently, a fourth and distantly related group of AFPs has been described^6^. The fungal genomes that encode more than one AFP include those from *Penicillium chrysogenum* and *Penicillium expansum*, which have one AFP belonging to each of three different classes^3^. In the case of the three *P*. *chrysogenum* AFPs (class A PAF, class B PgAfp and class C Pc-Arctin) their antifungal activity has been experimentally demonstrated^7–9^. Another representative of the *Penicillium* genus is the citrus postharvest pathogen *Penicillium digitatum*, a necrotrophic filamentous fungus that is highly specific for citrus fruits and produces very important economic losses worldwide^10,11^. The recent sequencing of its genome allowed the identification of several potential AFP-like proteins^10^, although further phylogenetic analyses confirmed the presence of only one *afp* gene (*afpB*) whose corresponding protein has been classified into class B and was called AfpB^3^.

The most characterized AFPs belong to class A: the AFP produced by *Aspergillus giganteus* ^12–15^, PAF from *P*. *chrysogenum* ^16–19^, and NFAP from *Neosartorya fischeri* ^20,21^. Unfortunately, *P*. *digitatum* AfpB has not been experimentally characterized so far due to the lack of protein detection in *P*. *digitatum* cultures even in constitutive expression strains that produce up to 1,000 times more *afpB* mRNA than the wild-type strain^3^. In any case, the tertiary structure of the putative AfpB was predicted by *in silico* molecular modelling, which allowed the identification of antifungal peptides based on the AfpB primary sequence and structure^22^.

There is a need for low cost and effective AFP-production platforms to produce these proteins at the scale and purity required for their different applications as new antifungal drugs. In this context, the *Pichia pastoris* expression system was applied to produce active recombinant *A*. *giganteus* AFP^23^, *P*. *chrysogenum* PAF and PAF mutants^16^ and *N*. *fischeri* NFAP and NFAP mutants^24,25^. Recently, a *P*. *chrysogenum*-based expression system for the production of AFPs was described, showing the feasibility of this approach for the overexpression of high amounts of PAF, PAF variants and NFAP^18,19^. The *P*. *chrysogenum* expression cassette consisted of the strong *paf* gene promoter, the *paf* pre-pro sequence (SP-pro sequence) for correct protein processing and secretion, and the *paf* gene terminator^8^. Moreover, this expression system was extended to heterologously express PAF in *P*. *digitatum* with similar yields to those obtained in *P*. *chrysogenum*, demonstrating the versatility of the system^19^.

In this study, we have produced the protein AfpB from *P*. *digitatum* for the first time using two different expression systems: i) homologous expression in *P*. *digitatum*, using the *P*. *chrysogenum-*based expression cassette containing the *paf* gene promoter and terminator sequences, and either the *paf* or the native *afpB* SP-pro sequence, and ii) heterologous expression in *P*. *pastoris*, using an inducible promoter and the yeast α-factor signal peptide sequence (α-factor SS). Recombinant AfpB has been successfully purified to homogeneity and structurally and functionally characterized.

## Results

### Recombinant production of AfpB in *P*. *digitatum* and *P*. *pastoris*

**Figure 1:**
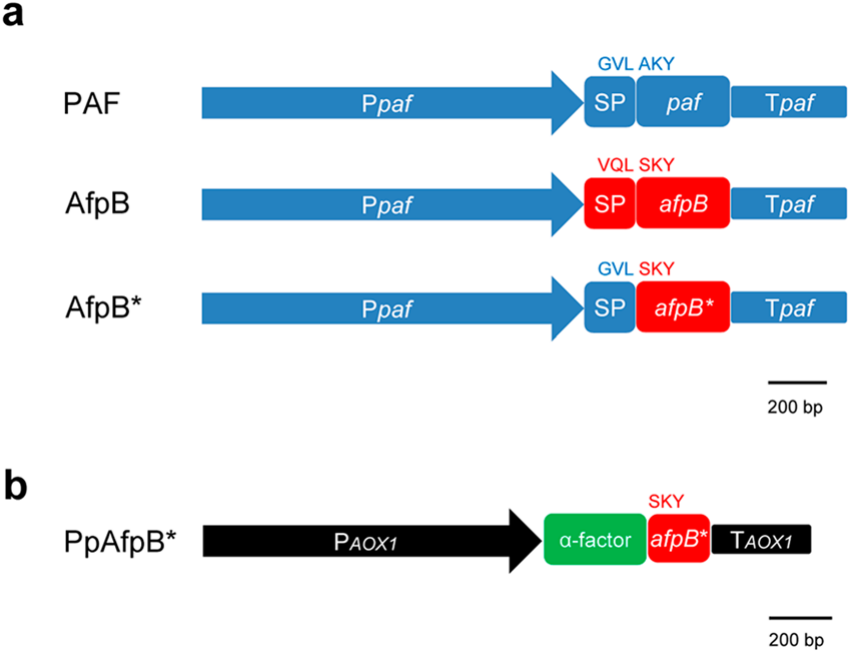
Schematic representation of the expression systems used to obtain AfpB producer strains. (**a**) The PAF diagram constitutes the schematic representation of the *P*. *chrysogenum*-based expression cassette; in blue: *paf* promoter (P*paf*), *paf* gene including the *paf* SP-pro sequence (SP), and *paf* terminator (T*paf*). Amino acids involved in the SP-pro peptide cleavage are coloured in blue. The AfpB diagram represents the genetic construction with the full-length AfpB coding sequence (in red) cloned under the control of the P*paf* and T*paf* sequences (in blue). Amino acids involved in the predicted SP-pro peptide cleavage are in red. The AfpB* diagram corresponds to the genetic construction with the *in silico* predicted AfpB coding sequence (afpB*) cloned under the control of the P*paf*, *paf* SP-pro sequence and T*paf*. (**b**) The PpAfpB* diagram represents the construction with the *in silico* predicted AfpB coding sequence (afpB*, in red) cloned under the control of the *AOX1* promoter for methanol-induced expression and terminator sequences (in black) and yeast α-factor signal sequence (α-factor SS) (in green).

We used the *P*. *chrysogenum*-based expression cassette^19^ to express AfpB in *P*. *digitatum* under the regulation of the strong *paf* promoter and terminator sequences (Fig. 1). In our previous work, the presence of the *paf* SP-pro sequence warranted the secretion of PAF, PAF variants and NFAP proteins into the supernatant independently of the *Penicillium* species used as cell factory. Here, in order to study the functionality of the native SP-pro sequence of the AfpB protein, two different transformation vectors with two expression cassettes for the *afpB* gene insertion in *P*. *digitatum* were constructed. Both constructions included the *paf* promoter and terminator sequences. However, in the first gene construction, the full-length *afpB* coding sequence (*afpB)* was cloned, while the second approach included the *in silico* predicted mature *afpB* coding sequence (afpB*) fused to the *paf* SP-pro sequence (Fig. 1a). After evaluation of positive transformants for protein production, one clone with the highest production of the recombinant AfpB protein for each construction was selected for further characterization. The selected producer strains were PDSG2441 for AfpB and PDSG3543 for AfpB*. The genetic modification of these strains was confirmed molecularly (Supplementary Fig. S1).

**Figure 2:**
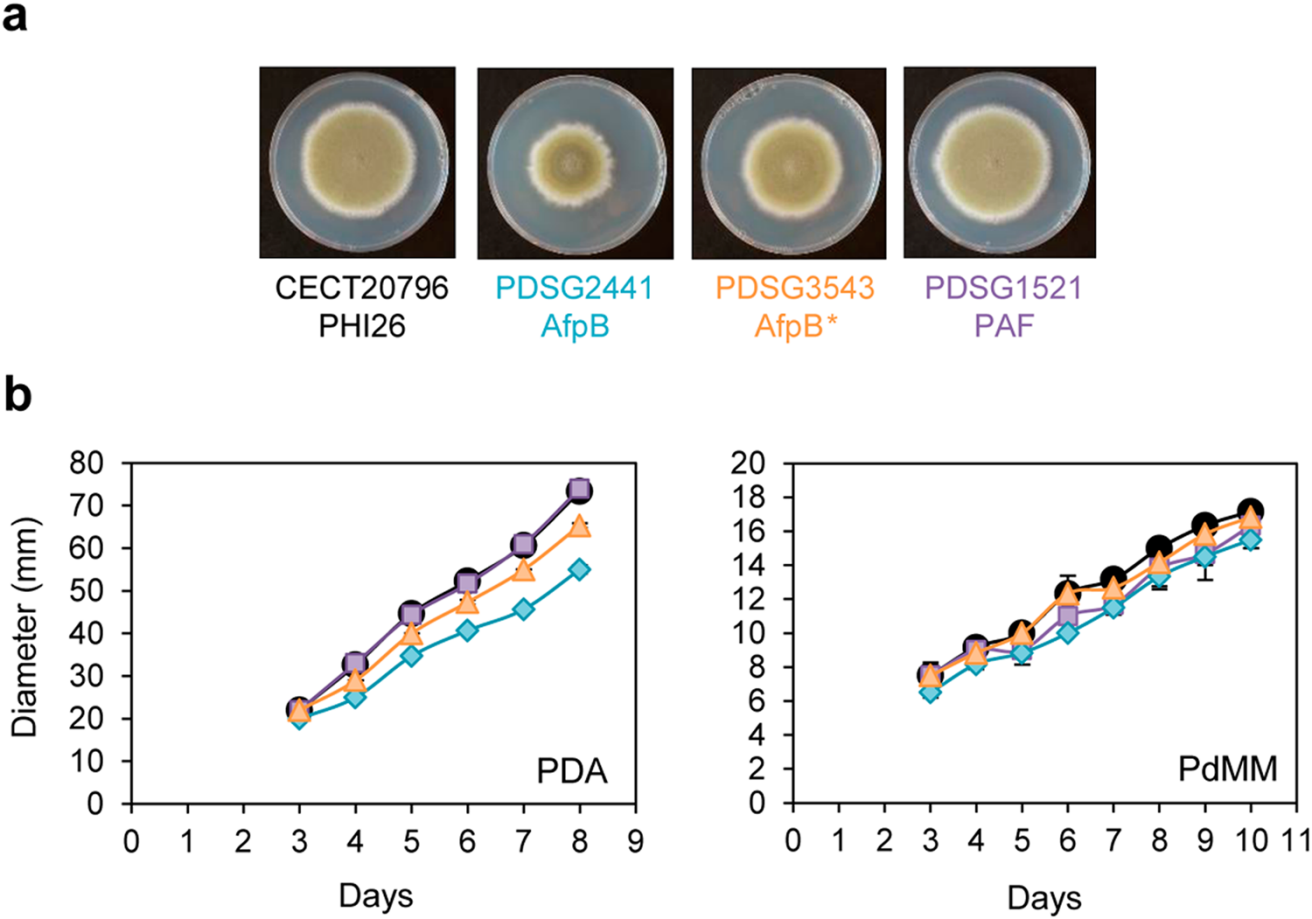
Growth of *P*. *digitatum* AfpB, AfpB* and PAF producer strains. (**a**) Colony morphology after 7 days of growth in PDA plates. (**b**) Growth on solid PDA and PdMM determined by the colony diameter from 3 to 10 days of growth at 25 °C. Plotted data are mean values ± s.d. of triplicate samples. The strains shown are the parental CECT 20796 (isolate PHI26) (black), the AfpB producer strains PDSG2441 (AfpB; blue) and PDSG3543 (AfpB*; orange), and the PAF producer strain PDSG1521 (purple).

The growth of the selected transformant strains in solid medium is shown in Fig. 2. Since the *P*. *chrysogenum* PAF was already expressed in *P*. *digitatum* ^19^, the PAF transformant strain (PDSG1521) was chosen as internal control in this study. AfpB transformants showed a moderate reduction of colony diameter on potato dextrose agar (PDA) plates (Fig. 2a,b), higher for the AfpB (PDSG2441) than for the AfpB* (PDSG3543) strain. However, the growth was indistinguishable from that of the parental strain in *P*. *digitatum* minimal medium (PdMM) (Fig. 2b). The PAF transformant did not show different phenotype to the parental strain in both media. The moderate reduction of growth shown in PDA plates for the transformants AfpB and AfpB* did not result in a difference in their pathogenicity and virulence shown during orange fruit infection (Supplementary Fig. S2).

Heterologous production of AfpB* was also addressed in *P*. *pastoris*. The genetic construct was designed for its gene inducible expression (*AOX1* promoter) and secretion of the corresponding protein (PpAfpB*, for AfpB* produced in *P*. *pastoris*) to the extracellular medium (Fig. 1b). With this purpose, afpB* cDNA was fused in frame to a shortened yeast α-factor SS, in which the Ste13 protease cleavage site was removed and the Kex2 protease cleavage site maintained. This modification was introduced to avoid the Ste13 partial cleavage of the secretion signal resulting in the accumulation of an inactive protein, as previously reported for the AFP from *A*. *giganteous* ^23^. The resulting construct was used to transform *P*. *pastoris* cells, and two independent transformant were selected for PpAfpB* production.

### Single-step cationic exchange chromatography allowed AfpB purification from *P*. *digitatum* and *P*. *pastoris* culture supernatants

**Figure 3:**
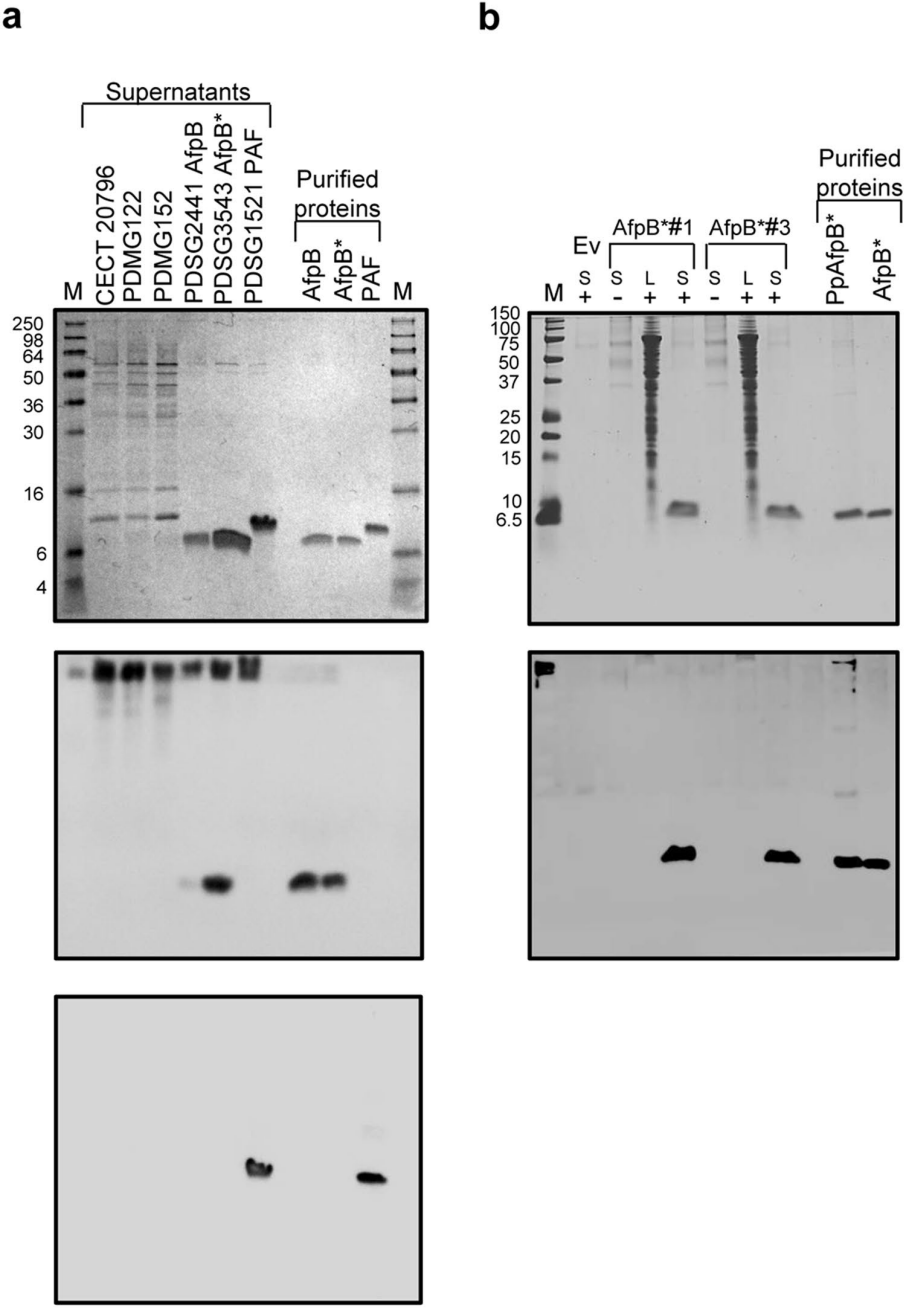
Analyses of supernatants of AfpB-producing strains and purified proteins by SDS-PAGE and Western blot. (**a**) *P*. *digitatum* supernatants (4–8 µg of total protein loaded per lane) and purified proteins (1 µg loaded per lane) visualized by Coomassie blue staining (top panel); M: SeeBlue® Pre-Stained Protein Standard, supernatants of wild-type strain CECT 20796, deletion strain PDMG122 Δ*afpB*, constitutive expression strain PDMG152 *afpB* ^c^, AfpB producer strain PDSG2441, AfpB* producer strain PDSG3543 and PAF producer strain PDSG1521. Immunoblot analyses of samples described above, using anti-PAFB (middle panel) and anti-PAF (bottom panel) antibodies. (**b**) *P*. *pastoris* supernatants (15 µg loaded per lane), cell lysates (50 µg loaded per lane) and purified proteins (1 µg loaded per lane) visualized by Coomassie blue staining (top panel). M: Precision Plus Protein Standard; AfpB*#1 and AfpB*#3: selected PpAfpB* producer colonies; Ev: control strain transformed with empty vector pPICZαA. S: supernatant; L: cell lysate; under non-induced conditions (−) or induced conditions ( + ). Immunoblot analyses of samples were performed as described above using anti-PAFB antibodies (bottom panel).

Selected clones for AfpB production in *P*. *digitatum* were grown in PdMM and, after clearing the culture broth from insoluble matter, the proteins in the supernatant were purified by one-step cation-exchange chromatography. Optimal production was achieved after 11 days of growth, and the protein amounts varied between 12 (AfpB) and 20 (AfpB*) mg/l. Both proteins eluted as a single chromatography peak at 0.25 M NaCl, and SDS-PAGE analysis revealed a single-protein band in both, having the same apparent molecular mass of approximately 6 kDa in agreement with the AfpB predicted molecular mass (6.46 kDa) (Fig. 3a, top panel). When comparing with the slightly smaller PAF (6.2 kDa), AfpB showed faster migration than expected. Anomalous migration in SDS-PAGE was also observed for NFAP^19^, PgAFP^9^ and other fungal AFPs^5^ and seems to be related to the extreme isoelectric points of these proteins^26^.

Two independent *P*. *pastoris* colonies were assayed to evaluate the PpAfpB* accumulation upon methanol induction at different time points. A polypeptide showing similar electrophoretic mobility to the AfpB produced in *P*. *digitatum* was observed in the supernatant of *P*. *pastoris* cells harbouring afpB* cDNA construct and grown under inducing conditions (Fig. 3b, top panel). The intensity of this band increased with time, reaching maximum levels at 48 hours of growth in methanol medium with strong aeration. This band was absent in the supernatant of cells grown under non-inducing conditions, as well as in cells transformed with the empty vector. It was not detected in the lysates of induced cells either, demonstrating its efficient secretion to the extracellular medium. The best producer colony was used to purify the secreted recombinant protein at large scale. The PpAfpB* was also easily purified to homogeneity by one-step cation-exchange chromatography (Fig. 3b, top panel) with yields of 1.2–1.4 mg/l.

### Immunodetection confirmed the absence of AfpB in the parental strain

Western blot analyses of 11-days old *P*. *digitatum* culture supernatants of different fungal strains and purified AfpB proteins were performed with antibodies against *P*. *chrysogenum* PAF^27^ and PAFB. PAFB is a protein from *P*. *chrysogenum* that is 88% identical to AfpB, and from which an antiserum was raised (unpublished). Culture supernatants of PDMG122 (null Δ*afpB*) and PDMG152 (constitutive *afpB* ^C^) were also included in the analyses as controls and to confirm previous absence of detection by Coomasie Blue staining^3^. Results indicated that both AfpB proteins and the corresponding supernatants reacted with anti-PAFB antibody (Fig. 2a, middle panel) while only purified PAF and the corresponding transformant strain PDSG1521 supernatant reacted with PAF antiserum (Fig. 2a, bottom panel). As expected, no protein band in the supernatant of the previous null transformant strain (Δ*afpB*) reacted with the anti-PAFB antibody. Remarkably, no immunoreaction was observed in the culture supernatant of either the parental strain (CECT 20796) or the constitutive *afpB* ^*c*^ transformant strain (PDMG152), fully demonstrating our previous conclusions on the absence of detection of any differential band or AfpB-like protein in those *P*. *digitatum* culture supernatants^3^.

Western blot analyses were also performed for the heterologously produced PpAfpB* protein with antibodies against PAFB (Fig. 3b, bottom panel). Supernatants from *P*. *pastoris* pPICZαA-AfpB* containing colonies grown under inducing conditions and purified PpAfpB* reacted with the antiserum while, as anticipated by Coomassie staining, no protein was detected in the non-induced supernatants or in cell lysates.

### Mass spectrometry revealed different processing of PAF and AfpB SP-pro sequence

**Figure 4:**
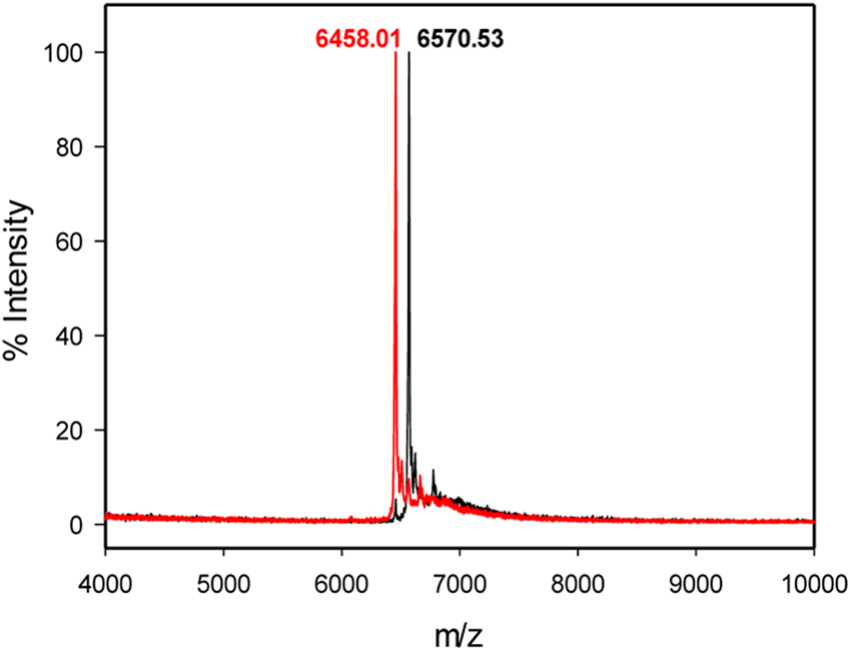
MALDI-TOF MS analyses. Data showing the isotopic average molecular mass (m/z) of the two recombinant protein variants AfpB* (red) and AfpB (black) produced in *P*. *digitatum*. The difference in the molecular mass fits with the extra leucine residue present at the N-terminus of the native protein AfpB.

Molecular mass of AfpB proteins purified from *P*. *digitatum* was also determined by matrix-assisted laser desorption/ionization–time-of-flight mass spectrometry (MALDI-TOF MS) analysis. Single peaks corresponding to average masses of 6570.53 and 6458.01 Da were detected for AfpB and AfpB*, respectively (Fig. 4). The experimental mass of AfpB* is consistent with the calculated theoretical mass of the oxidized protein predicted after cleavage from the PAF SP-pro sequence (6456.23 Da), indicating the presence of three intra-molecular disulphide bonds and the absence of other post-translational modifications. Interestingly the average mass detected for AfpB revealed the presence of an extra leucine residue at the N-terminus end of the protein (calculated theoretical mass 6575.39 Da) suggesting a different cleavage of the native AfpB SP-pro sequence.

The MS corresponding to PpAfpB* revealed one main signal of 6455.20 Da (Supplementary Fig. S3) in accordance with the calculated molecular mass of the oxidized protein form and the proper processing of the yeast α-factor SS. An additional minor signal of 7104.54 Da that did not correspond to any potential variable N-terminus was detected.

### Electronic circular dichroism spectroscopy assays revealed incomplete refolding capability of the AfpB* variant

**Figure 5:**
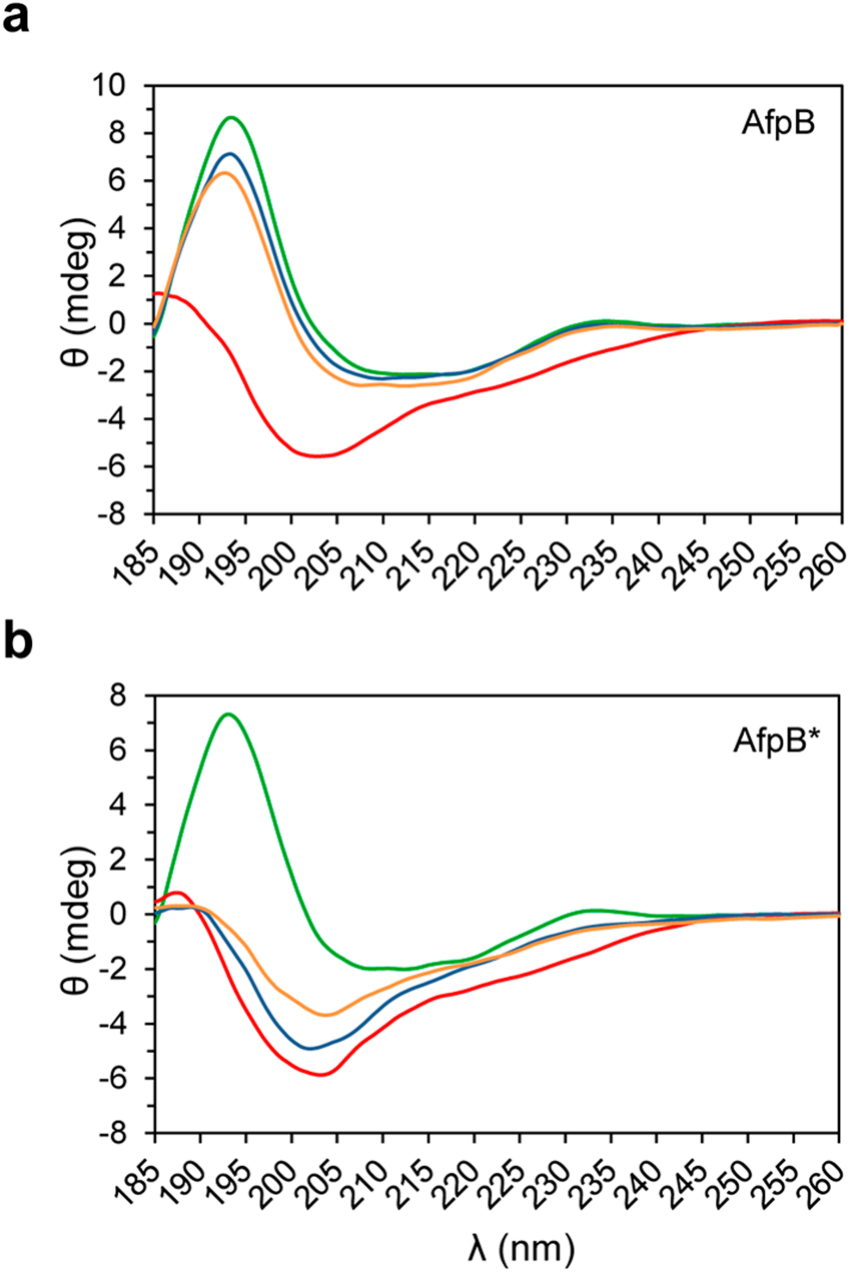
ECD spectra of the two recombinant protein variants produced in *P*. *digitatum*. (**a**) AfpB, and (**b**) AfpB* recorded at 25 °C (green), 95 °C (red), and at 25 °C immediately (blue), and 72 h (orange) after cooling from 95 °C to 25 °C.

Electronic circular dichroism (ECD) spectroscopy was used to determine protein conformation and proper folding of both *P*. *digitatum* AfpB and AfpB* variants. The ECD spectra recorded at 25 °C were nearly identical for both variants (Fig. 5 and Supplementay Fig. S4), and similar to those recorded for PAF, NFAP^19^ and other disulphide bridged, β-structured proteins^28^. The spectra had two maxima at 195 and 229 nm. The maximum at 229 nm was mainly attributed to the presence of disulphide bridges while the maximum centred at 195 nm reflected contributions from both β-pleated conformation and the electronic transitions of disulphide bridges. Spectra measured at 95 °C reflected the loss of ordered structure in both AfpB variants. After cooling back to 25 °C, the native fold of AfpB was restored completely and almost immediately (Fig. 5a). In contrast, a slow structural reorganization of AfpB* took place with incomplete refolding even after 72 h (Fig. 5b). This observation may be attributed to the absence of the leucine residue at the N-terminus of this protein variant.

### AfpB was highly active against *Penicillium* species including *P*. *digitatum*

**Figure 6:**
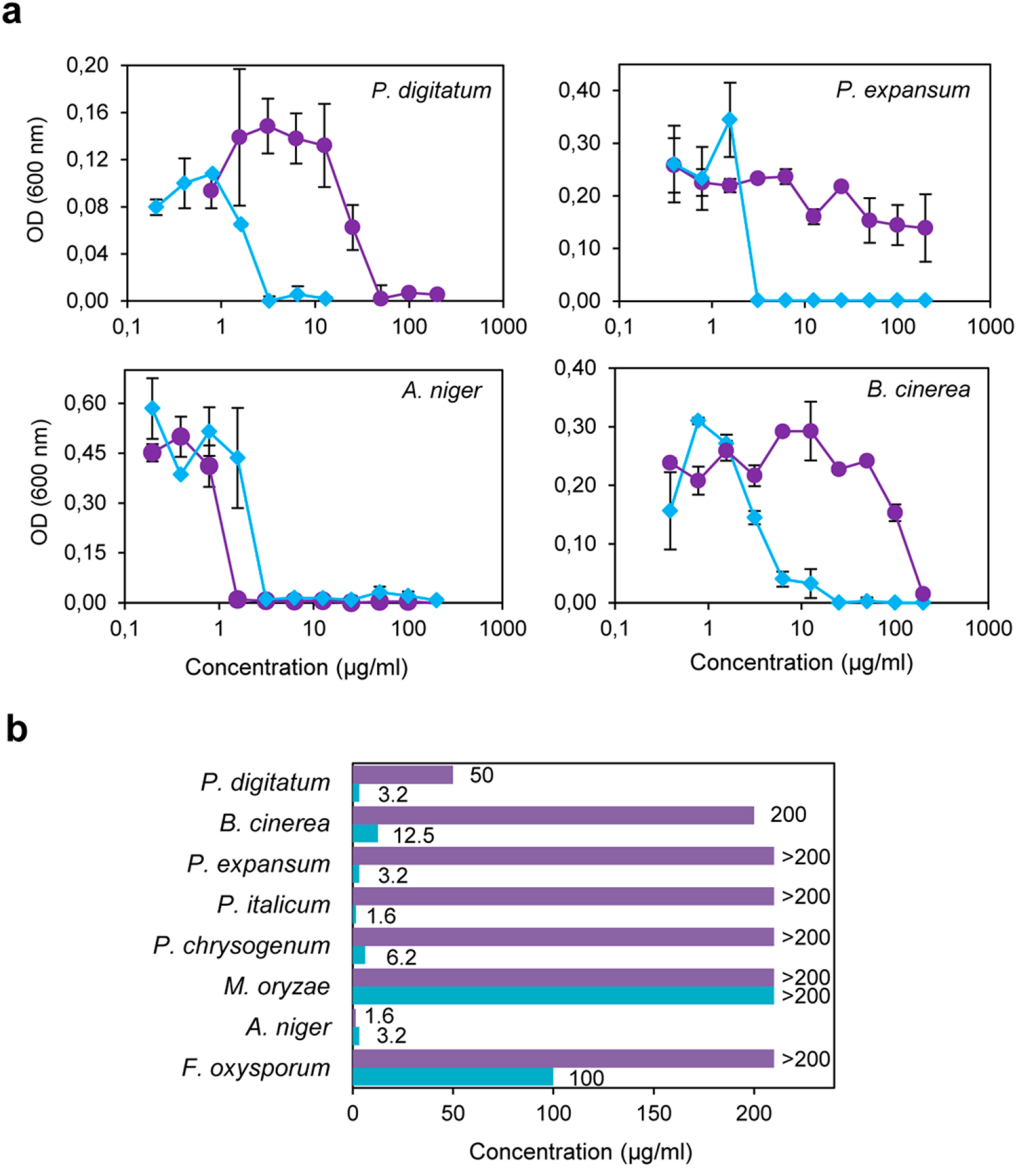
*In vitro* inhibitory activity of AfpB and PAF against filamentous fungi. (**a**) Dose-response curves comparing the antifungal activity of PAF (purple circles) and AfpB (blue diamonds) against *P*. *digitatum*, *P*. *expansum*, *A*. *niger*, and *B*. *cinerea*. Dose-response curves show mean ± s.d. OD_600_ of triplicate samples after 72 h of static incubation at 25 °C, except for *A*. *niger*, which was incubated at 37 °C. (**b**) MIC values (µg/ml) of AfpB and PAF against all the fungi tested.

Both AfpB protein variants, and PAF as an internal control, were tested for their antimicrobial activity towards *Escherichia coli*, *Saccharomyces cerevisiae* and a selection of filamentous fungi that include, in addition to the *P*. *digitatum* parental strain, several plant pathogens such as the citrus fruit specific *Penicillium italicum*, the main postharvest pathogen of pome fruit *P*. *expansum*, the polyphagous *Botrytis cinerea*, the rice blast fungus *Magnaporthe oryzae*, and the soilborne plant pathogen *Fusarium oxysporum*. The PAF producer *P*. *chrysogenum* strain, and a strain from *Aspergillus niger* which is particularly sensitive to the PAF protein were also tested. No differences in antimicrobial activity were observed among AfpB, AfpB* and PpAfpB* produced in either *P*. *digitatum* or *P*. *pastoris* (Supplementary Fig. S5). Therefore, only results from AfpB produced in *P*. *digitatum* are shown. AfpB was inactive against *E*. *coli* and *S*. *cerevisiae* at the highest concentration tested (200 µg/ml). AfpB showed antifungal activity and inhibited the growth of all fungi tested, with the exception of *M*. *oryzae* (Fig. 6). The minimum inhibitory concentration (MIC) values varied from 1.6 µg/ml in *P*. *italicum* to 100 µg/ml in *F*. *oxysporum*. The three plant pathogenic *Penicillium* species tested were considerably sensitive to the protein, including its producer strain. With the exception of the PAF-sensitive *A*. *niger*, AfpB showed higher antifungal activity than PAF, with MIC values at least one order of magnitude lower.

### AfpB showed high protease and thermal resistance

The sensitivity of AfpB to proteolytic digestion was tested using proteinase K and evaluating the residual antifungal activity against *P*. *digitatum*. Preincubation of AfpB and AfpB* with proteinase K did not decrease the antifungal activity of any of both protein variants (Supplementary Fig. S6), pointing out the high protease resistance of these antifungal proteins.

The incomplete refolding of AfpB* found with the ECD analyses allowed us to test and determine the effect of heat treatment and folding on the antifungal activity. After heat treatment at 95 °C for 5 min and cooling back to 25 °C (mimicking the ECD conditions) the antifungal activity of both protein variants was practically equal and comparable to that observed without treatment (Supplementary Fig. S7), suggesting that native AfpB refolding is not necessary for its antifungal activity.

**Figure 7:**
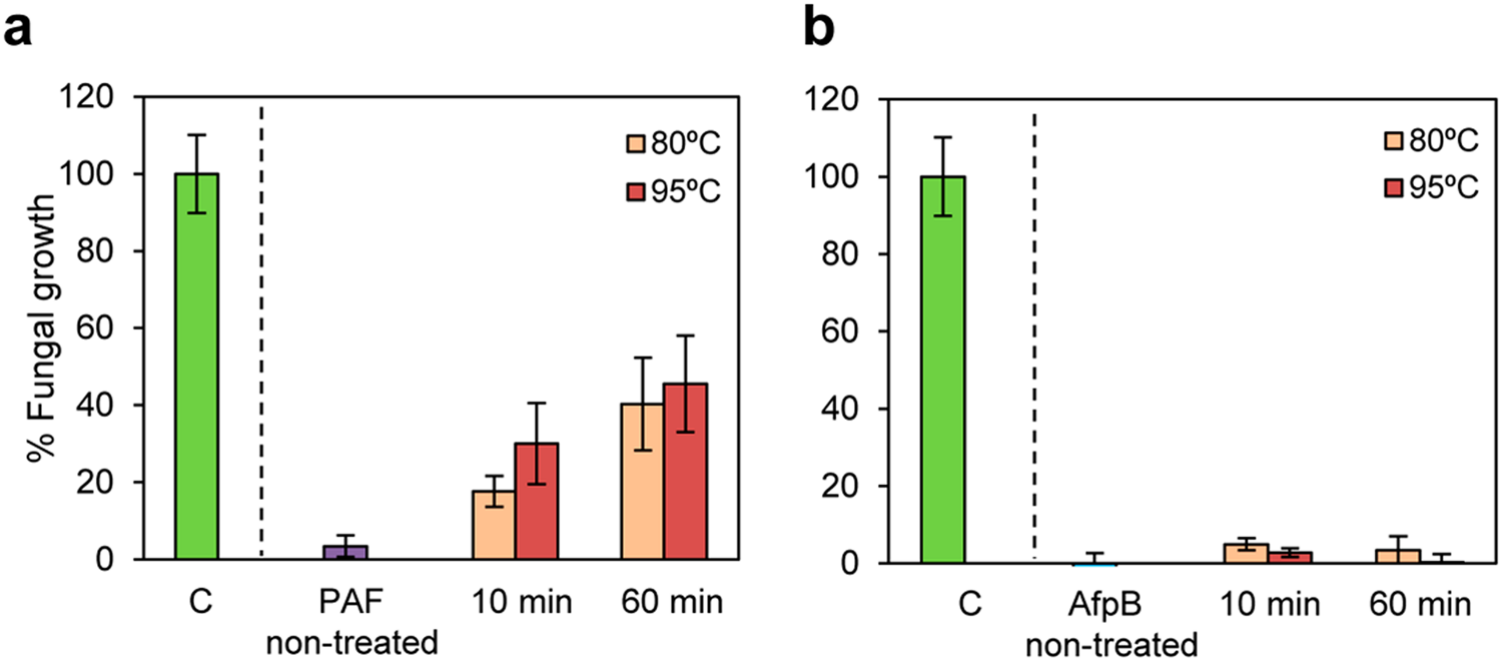
Effect of heat treatment on the antifungal activity of PAF and AfpB. PAF (**a**) and AfpB (**b**) (100 and 6.5 µg/ml, respectively) were exposed to 80 and 95 °C for 10 and 60 min, respectively. Values represent the percentage of growth (%) of *P*. *digitatum* in the presence of non-treated and treated protein compared to *P*. *digitatum* in the absence of protein (control, **C**). Data show mean ± s.d. OD_600_ of triplicate samples.

Further experiments were conducted to compare the thermal stability of PAF and AfpB. After heat treatment at 80 °C or 95 °C for 10 and 60 min and cooling back to 25 °C, the antifungal activity of PAF and AfpB were tested against *P*. *digitatum*. Fungal conidia were exposed to twofold MIC concentrations of heat-treated proteins (100 µg/ml for PAF, Fig. 7a, and 6.5 µg/ml for AfpB, Fig. 7b). The antifungal activity of PAF against *P*. *digitatum* at that concentration resulted in a growth reduction of 70–80% after 10 min of heat treatment at 95 °C and 80 °C, respectively in comparison with the untreated control (>95%). After 60 min of heat treatment, PAF showed a *P*. *digitatum* growth reduction of 60% at 80 °C and 55% at 95 °C. In the same conditions, AfpB showed total inhibition of the fungus growth (>95%, comparable to the non-treated control), demonstrating that AfpB is more resistant to heat treatment than PAF.

### AfpB showed no haemolytic activity against human red blood cells

Haemolytic activity assays are performed in order to determine the cytotoxicity of specific proteins and peptides against eukaryotic cells by their ability to lyse human red blood cells (RBCs)^29^. In this study, we determined the haemolytic activity of both AfpB variants at different concentrations from 1 to 100 μM (approximately 200 times the MIC against *P*. *digitatum*). In these experiments, PAF and melittin from honeybee^30^ were included as negative and positive controls, respectively. Since the haemolytic properties of cationic peptides may show ionic strength dependence, haemolytic assays were conducted not only with a high ionic strength phosphate NaCl buffer (PBS) but also with a low ionic strength isotonic glucose phosphate buffer (PBG)^29^.

**Figure 8:**
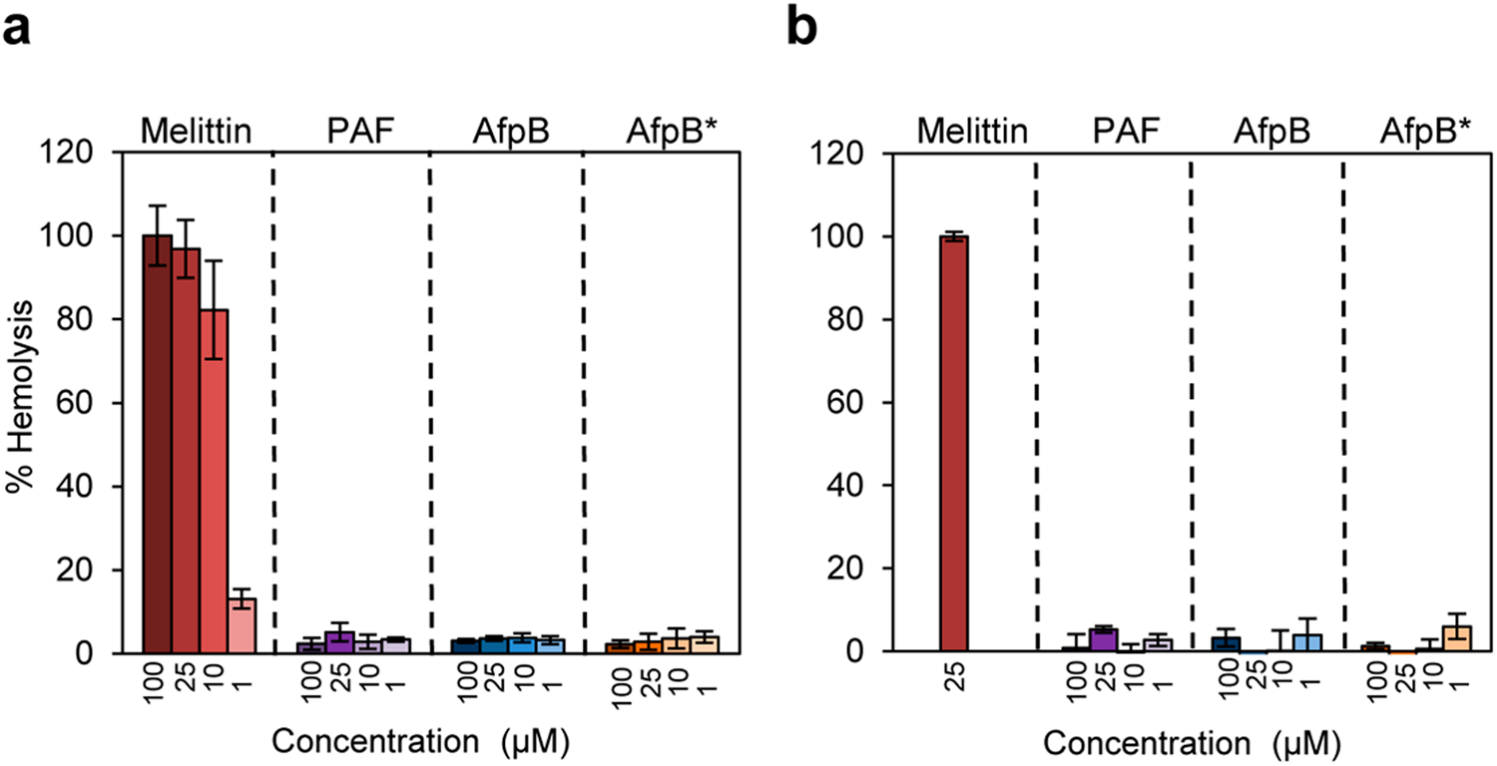
Haemolytic activity of the two AfpB variants and PAF. Analyses were conducted in PBS (150 mM NaCl) (**a**), and in PBG (250 mM glucose) (**b**). Proteins were used at the concentrations indicated (from 1 to 100 µM). The cytolitic peptide melittin was included for comparison. The haemolytic activity is given as the mean ± s.d. of the percentage of human RBCs haemolysis (three replicates), as compared with the positive control in the presence of the detergent Triton X-100 (regarded as 100% haemolysis).

As expected, the haemolytic peptide melittin caused 100% haemolysis at the highest concentration tested and around 80% at 10 μM (Fig. 8). By contrast, none of the AFPs showed haemolytic activity at any of the concentrations tested, neither in the presence of NaCl as in PBS (Fig. 8a) nor glucose (Fig. 8b), with values close to the control where no protein was added.

## Discussion

In this study, we described the biotechnological production and characterization of AfpB, the only AFP encoded in the phytopathogenic fungus *P*. *digitatum*. This protein is a new member of AFPs grouped into the class B phylogenetic cluster^3^. For the first time, AfpB was produced, detected and purified from culture supernatants of recombinant *P*. *digitatum* and *P*. *pastoris* strains. The homologous production of AfpB in *P*. *digitatum* with the *paf*-derived expression cassette resulted in high yields around 12–20 mg protein/l, whereas the heterologous production in *P*. *pastoris* resulted in yields 10-fold lower (1.2–1.4 mg/l). Both approaches allowed one-step purification of fully active AfpB, and the purified protein from the two systems showed the same antifungal potency. Our results demonstrate that both filamentous fungi and yeasts are good production platforms for AFPs. The homologous system appears as the most efficient system reaching high yields; whereas the heterologous system has the advantage of time reduction since optimal production was achieved after 11 days of growth in *P*. *digitatum* while only 2 days were needed with the *P*. *pastoris* system.

Previously, three other class B AFPs were characterized: Anafp from *A*. *niger* ^31^, PgAFP from *P*. *chrysogenum* ^32^ and MAFP1 from *Monascus pilosus* ^33^. They show an amino acid identity with *P*. *digitatum* AfpB of 79, 88, and 83%, respectively, much higher than the percentage of identity shared between AfpB and the class A PAF from *P*. *chrysogenum*, with only 33% identity^3^. The three of them were successfully isolated from the culture supernatants of the corresponding native and producer fungus. Notably, the concentration of purified PgAFP obtained from *P*. *chrysogenum* CECT 20922, which was isolated from cured meat, was up to 700 µg/ml^32^. Also, the class A PAF and AFP are secreted in large amounts by *P*. *chrysogenum* ^17^ and *A*. *giganteus* ^13^, respectively. However, previous attempts to detect AfpB in *P*. *digitatum* culture supernatants failed, even in *P*. *digitatum* transformants for a*fpB* constitutive expression under the strong *gpdA* promoter from *A*. *nidulans* ^3^. Remarkably, those transformant strains showed a drastic reduction of axenic growth, abnormal hyphal morphology and delayed conidiogenesis^3^. Here, we have confirmed with immunodetection that AfpB is not produced by any of the previous strains. By contrast, *afpB* expression under the regulation of the strong *paf* promoter and the corresponding *paf* terminator warranted the production and secretion of the protein at high levels, concomitant with only a moderate reduction of axenic growth. We showed that AfpB production is similar in the presence of either the *paf*- or the *afpB*-specific SP-pro sequences, indicating that the impossibility to detect AfpB in either the parental or constitutive strain cultures is not due to the native AfpB SP-pro peptide, but to the regulatory sequences. The *P*. *chrysogenum paf* promoter has been demonstrated to have greater efficiency than the *A*. *nidulans gpdA* constitutive promoter used for the expression of reporter genes in *P*. *chrysogenum* ^34^, however, the approximately 2-fold difference in efficiency cannot account for the absence of AfpB accumulation when the *gpdA* promoter was used. In addition, differences in 5′ untranslated regions (UTRs) in the mRNA have been demonstrated to play an important role in determining the translation efficiency of proteins in filamentous fungi^35^. Nevertheless, AfpB production was also achieved in the *P*. *pastoris* expression system under the control of the *AOX1* promoter using the yeast α-factor SS. Additional studies are required to elucidate the exact mechanism underlying the problematic of native *afpB* translation and protein accumulation in *P*. *digitatum*.

In accordance with the antimicrobial profile of native AFPs that possess antifungal but not antibacterial activity^1,17^, AfpB displayed antifungal activity against selected filamentous fungi and it was inactive against bacteria (*E*. *coli*) or yeast (*S*. *cerevisiae*). However, the homologous protein AnAFP from *A*. *niger* inhibited the proliferation of *Candida albicans* and *S*. *cerevisiae* ^31^ whereas the other two class B representatives did not show any effect on different bacteria and yeast species^32,33^. Recently a new AFP from *N*. *fischeri* named NFAP2 proved to be highly effective against targeted yeasts including clinically relevant *Candida* species^6^. NFAP2, which seems to be the first member of a new, phylogenetically distinct fourth group among AFPs, showed MIC values in the range of 0.2–1.5 µg/ml. Potential AfpB anti-yeast activity against non-laboratory strains and/or pathogenic isolates deserves future research.

Until this study it was assumed that AFPs are not active against the producer fungus, and it has been speculated that fungi possess innate sensing or defense systems which enable them to discriminate between AFPs from self or non-self origin^36^. Whether the function of fungal AFPs is mainly defensive or associated with fungal growth and development is still controversial. Null mutation of the *afpB* gene in *P*. *digitatum* did not affect spore production, growth or virulence^3^, contrarily to that described for the null mutation of the *paf* gene in *P*. *chrysogenum* that severely affected asexual development, spore production and associated gene regulation^37^. Recently, novel functions related to nutrient recycling during starvation, autophagy, and development for the AnAFP produced by *A*. *niger* have been proposed^36^. It is very tempting to speculate that AFPs from different classes do have distinct biological functions in the producer fungus and also distinct mechanisms of antifungal action. This seems to be the case in the AfpB/PAF couple, according to the different phenotypes of the corresponding null mutants but also to the different activity profiles of both proteins against *P*. *digitatum*, *P*. *expansum* or *A*. *niger*.

AfpB is highly active against the own producer *P*. *digitatum* strain when added exogenously to the culture media (MIC = 3.2 µg/ml; Fig. 6), which is not the case of PAF against *P*. *chrysogenum* (MIC > 200 µg/ml). However, the fungal strain engineered to express the *afpB* gene produces high amounts of AfpB without deleterious effect to the axenic growth or pathogenicity of *P*. *digitatum*. This fungus is also susceptible to PAF protein (MIC = 50 µg/ml) but *P*. *digitatum* engineered strains were also able to produce PAF in high quantities^19^. In contrast, the heterologous expression of the *nfap* gene in a NFAP-sensitive *A*. *nidulans* strain only permitted the production of low amounts of protein (approximately 1.7 mg/l), but it provoked reduced hyphal growth and delayed and abnormal germination^38^. Other fungal AFPs such as AnAFP and *A*. *giganteous* AFP have been described as only moderate-active towards the producer strains^36^ whereas the class B PgAFP was not active against its producing strain *P*. *chrysogenum* CECT 20922 while it showed antifungal activity against another *P*. *chrysogenum* strain, pointing out strain-dependent activity^32^.

AfpB is unique for its very high antifungal activity against filamentous fungi that include the fungus from which the gene was identified. Our side-by-side experiments demonstrate that AfpB has higher levels of antifungal efficacy when compared to PAF (Fig. 6). With respect to other class B proteins already characterized, AnAfpB showed similar antifungal activity against *F*. *oxysporum* ^31^, and PgAfpB was as well active against other *P*. *expansum* and *A*. *niger* strains^32^. The activity of AfpB against the *Penicillium* species tested deserves to be emphasized. All of them were highly sensitive to AfpB: MIC values in the range of 1.6–6.25 µg/ml that is equivalent to 0.25–1 µM. Remarkably, current fruit losses due to *Penicillium* decay are very important in agriculture since *Penicillium* species reproduce very rapidly and their spores are ubiquitous in the atmosphere, facilitating the dissemination on fruit surfaces. *P*. *digitatum and P*. *italicum* are the main causal agents of the citrus green and blue mold diseases, respectively^10^. On the other hand, *P*. *expansum*, the causal agent of the blue mold disease, is one of the most important pathogens of pome fruits, causing serious crop losses worldwide^11^. Unfortunately, there are only a few drugs available for the effective treating of fungal infections, and the development of resistance against fungicides used in agriculture is increasingly alarming. Thus, the use of AfpB as a powerful alternative in the control of pathogenic fungi could be of further interest.

In a previous work, we identified two cysteine-containing cationic peptides, PAF112 and PAF118, derived from two surface-exposed loops in AfpB, with moderate antifungal activity against *P*. *digitatum* with a MIC value of 64 µM^22^. Now our results show that AfpB is about 100 times more active than the peptides derived from its sequence suggesting that additional motifs/sequences in the protein are required to achieve full activity.

As generally described for fungal AFPs^16^, AfpB showed a remarkable ability to withstand protease and thermal degradation. It has been assumed that the compact tertiary structure stabilized by disulphide bridges of AFPs favours protein stability. Although the tertiary structure of AfpB has not yet been determined, the predicted AfpB structure by *in silico* modelling showed a similar tertiary structure to those reported for A. *giganteous* AFP^12^ and *P*. *chrysogenum* PAF^16,39^. In the present study ECD spectroscopic measurements, consistent with the previous modelling, suggested the presence of disulphide bridges and a β-pleated conformation, and proved that both AfpB variants have the same structural elements as other fungal AFPs^19,24^. Our data also demonstrate that the lack of the leucine residue at the N-terminal end of AfpB* does not affect neither the antifungal activity nor the protease and thermal tolerance, although this protein variant showed incomplete refolding in the conditions tested. Moreover, the demonstration that the heat treatment which denatures AfpB*, mostly irreversibly, does not impair antifungal efficacy indicates that protein folding is not critical for the antifungal activity.

Finally, our results suggest that AfpB can be preliminary regarded as safe since it did not show haemolytic activity against human RBCs even in assays conducted at low ionic strength isotonic conditions, which are considered more sensitive for detecting the haemolytic activity of cationic peptides^29^.

In summary, this study identifies AfpB from *P*. *digitatum* as a highly active antifungal protein against filamentous fungi, including the own producer fungus. This new member of class B AFPs was produced for the first time in two eukaryotic cell factories, the fungus *P*. *digitatum* and the yeast *P*. *pastoris*. Both expression systems allowed proper protein folding, efficient production and single-step purification from culture supernatants. The remarkable stability, absence of haemolytic activity and high levels of antifungal efficacy against filamentous fungi, especially *Penicillium* species, suggest the potential use of AfpB as an antifungal agent.

## Methods

### Strains, media and culture conditions

The parental isolate *P*. *digitatum* CECT 20796 (PHI26)^10^ and all of the transformants were cultured on PDA plates for 7–10 days at 25 °C. To analyse the growth on solid medium, 5 µl of conidial suspension (5 × 10^4^ conidia/ml) were deposited on the centre of PDA and PdMM plates^19^, and the diameter of growth was monitored daily from 3 to 10 days. For AfpB production, strains were inoculated at a concentration of 10^6^ conidia/ml in 500 ml of PdMM and incubated at 25 °C with shaking for 14 days. For the antimicrobial assays, *B*. *cinerea* CECT 2100, *F*. *oxysporum* 4287, *P*. *expansum* CMP1, *P*. *italicum* CECT 2294, *P*. *chrysogenum* Q176, and *M*. *oryzae* PR9 were incubated at 25 °C, *E*. *coli* JM109 and *A*. *niger* CBS 120.49 at 37 °C, and *S*. *cerevisiae* BY4741 at 30 °C. For the fungal transformation, vectors were propagated in *E*. *coli* JM109 grown in Luria Bertani (LB) medium supplemented with 100 µg/ml ampicillin or 75 µg/ml kanamycin. *Agrobacterium tumefaciens* AGL-1 strain was grown in LB supplemented with 20 µg/ml rifampicin at 28 °C. *P*. *pastoris* X-33 wild-type strain was cultured on yeast extract peptone dextrose (YPD) medium at 28 °C.

### Generation of the *P*. *digitatum* AfpB producer strains

To generate the *P*. *digitatum* AfpB producer strains, two different genetic approaches were performed. The first approach included the full-length AfpB coding sequence (*afpB)* cloned under the control of the *paf* gene promoter and terminator sequences from *P*. *chrysogenum*. The second approach included the *in silico* predicted mature AfpB coding sequence^3^ (afpB*) cloned under the control of the *paf* gene promoter, *paf* SP-pro sequence and the *paf* gene terminator (Fig. 1a). The specific primers used to generate the *P*. *digitatum* AfpB producer strains are described in Supplementary Table S1 and Supplementary Fig. S1a. The *afpB* gene was amplified from *P*. *digitatum* CECT 20796 genomic DNA, while the *paf* gene promoter, SP-pro and terminator sequences were generated by PCR amplification of the fragments from the vector pSK275*paf* ^19^. PCR reactions were performed using AccuPrime High-Fidelity polymerase (Invitrogen) and the constructs verified by Sanger DNA sequencing. The two different DNA constructions were generated by Fusion PCR^40^ and cloned into the pGEM-T® Easy vector system (Promega) from where they were excised with *Xma*I and *Xba*I and inserted into the digested binary vector pBHt2^41^ containing the hygromycin resistant cassette (*hph*) used as positive selection marker. The binary vectors were transformed into *A*. *tumefaciens* AGL-1, and the fungal transformation of *P*. *digitatum* parental strain was performed by *A*. *tumefaciens-*mediated transformation (ATMT)^41,42^. Positive transformants were confirmed by PCR amplification of genomic DNA^43^ (Supplementary Fig. S1; Supplementary Table S1).

### Generation of the *P*. *pastoris* AfpB producer strains

To generate the *P*. *pastoris* AfpB producer strain, the *P*. *digitatum* cDNA encoding the predicted AfpB protein sequence (afpB*) was obtained as previously described^43^ and amplified by PCR using the primers MO3 and MO4 (Supplementary Table S1). The afpB* cDNA sequence was inserted into the *Xho*I and *Xba*I digested pPICZαA plasmid (Invitrogen) carrying *AOX1* promoter for methanol-induced expression of afpB* in *P*. *pastoris*. The afpB* cDNA was inserted in frame with a modified version of the yeast α-factor SS, lacking the Ste13 cleavage sites, but including the Kex2 signal cleavage site (Fig. 1b). Constructs were verified by Sanger DNA sequencing. The digested plasmid pPICZαA-*afpB* with *Avr*II was used to transform *P*. *pastoris* X-33 cells by electroporation. After 5 min of incubation at 4 °C, cells were subjected to a pulse (1.5 kV, 200 Ω) in 0.2-cm cuvettes and were immediately diluted with 1 ml of ice-cold 1 M sorbitol and plated on YPD medium containing 1 M sorbitol and 100 µg/ml zeocin. Plates were incubated for 3–4 days at 28 °C. Two independent colonies were selected (#1 and #3) and used for PpAfpB* production. Transformant cells were grown in buffered minimal glycerol medium BMG (0.1 M potassium phosphate buffer, pH 6; 1.34% yeast nitrogen base; 4 × 10^–5^% biotin; 1% glycerol), and transferred to BMM (buffered minimal medium with 0.5% methanol instead of glycerol as carbon source) for afpB* induction.

### Protein production and purification

AfpB variants were purified from the supernatants of *P*. *digitatum* transformant strains (AfpB and AfpB*) and *P*. *pastoris* positive transformants (PpAfpB*). Cell-free supernatants of *P*. *digitatum* grown on PdMM for 11 days or *P*. *pastoris* grown on BMM for 2 days were collected by centrifugation and dialyzed (2 K MWCO,) against 20 mM phosphate buffer pH 6.6. Dialyzed solutions were applied to an AKTA Purifier system equipped with a 6 ml RESOURCE S column (GE Healthcare) for *P*. *digitatum* supernatants or a 5 ml HiTrap SP HP column (GE Healthcare) for *P*. *pastoris* supernatants, equilibrated in phosphate buffer. Proteins were eluted with a linear NaCl gradient from 0 to 0.5 M in the same buffer.

Protein containing fractions were pooled, dialyzed against Milli-Q water and lyophilized. Protein concentrations were determined spectrophotometrically (A_280_) considering the molar extinction coefficient (ε280 = 0.52). Purification was monitored by SDS-PAGE^44^ using SDS-16% polyacrylamide gels calibrated with prestained protein size-standard SeeBlue® (ThermoFischer Scientific) or Precision Plus Protein Standards (Bio-Rad) and Coomassie stained.

### Western blot

Total proteins from supernatants, lysates and purified AfpB variants were separated by SDS-16% polyacrylamide gels and transferred to Amersham Protran 0.20 µm NC nitrocellulose transfer membrane (GE Healthcare). Protein detection was accomplished using anti-PAFB (unpublished) and anti-PAF^27^ antibodies diluted 1:1000 and 1:2000, respectively. As secondary antibody, ECL NA934 horseradish peroxidase donkey anti-rabbit (GE Healthcare) was used and chemiluminescent detection was performed with ECL™ Select Western blotting detection reagent (GE Healthcare) using a LAS-1000 instrument (Fujifilm). The experiments were repeated twice.

### MALDI-TOF-MS

The mass of the purified proteins was analysed on a 5800 MALDI TOF/TOF (AB Sciex) in positive linear mode (1500 shots every position) in a range of 2000–20000 m/z. The analysis was carried out in the proteomics facility of SCSIE University of Valencia (Spain).

### ECD spectroscopy

ECD spectroscopic measurements were performed in the 185–260 nm wavelength range (far–UV) to determine the secondary structure and examine the structural stability of the two AfpB variants. Protein samples were dissolved in pure H_2_O at approximately 0.1 mg/ml concentration and measured in a 0.1 cm path-length quartz cuvette^19^. The spectra are accumulations of 10 scans, from which the similarly recorded spectrum of H_2_O was subtracted. Ellipticity data were given in mdeg units.

### Protein stability assays

To investigate thermal stability, proteins (400 µg/ml) were dissolved in 10 mM 3-(*N-*morpholino)-propanesulfonic acid (MOPS) pH 7 and were incubated at 80 °C and 95 °C for 5, 10 and 60 min. Proteins were cooled back to 25 °C and directly used to determine the residual antifungal activity.

Proteolytic digestion assays were performed as previously described^45^ with some modifications. Briefly, proteins (400 µg/ml) were dissolved in 10 mM MOPS pH 7 and digested with 100 µg/ml of recombinant proteinase K (2 U/mg; Sigma-Aldrich) at 30 °C with shaking. Aliquots were withdrawn at 20 h of incubation and immediately heated at 80 °C for 10 min. Treated proteins were used to perform antifungal activity assays.

### Antimicrobial activity assays

Growth inhibition assays were performed in 96-well, flat-bottom microtiter plates in a total volume of 100 µl. Fifty µl of fungal conidia (5 × 10^4^ conidia/ml) in 1/10 diluted potato dextrose broth (PDB) containing 0.02% (w/v) chloramphenicol were mixed in each well with 50 µl of twofold concentrated protein from serial twofold dilutions (final concentrations from 0.2–200 µg/ml). Samples were prepared in triplicate. Plates were statically incubated for 96 h at 25 °C. Growth was determined every 24 h by measuring the optical density (OD) at 600 nm using a Fluostar Omega plate spectrophotometer (BMG labtech), and the OD_600_ mean and standard deviation (s.d.) were calculated. Dose-response curves were generated from measurements after 72 h. These experiments were repeated at least twice. MIC is defined as the protein concentration that completely inhibited growth in all the experiments conducted.

### Haemolytic assays

The haemolytic activity of the proteins was determined in a 96 round-bottom microtiter plate on human 1:40 diluted RBCs as previously described^29,46^ with some modifications. Briefly, RBCs were harvested by centrifugation for 15 min at 100 × g and washed three times in 35 mM PBS (pH 7, 150 mM NaCl) or PBG (250 mM glucose as osmoprotectant). One hundred µl of twofold protein concentration were mixed with 100 µl of RBCs in triplicate. Plates were incubated for 1 h at 37 °C and centrifuged for 5 min at 300 × g. Supernatants (100 µl) were transferred to a new microtiter plate and the absorbance was measured at 415 nm.

No haemolysis and 100% haemolysis were determined in controls with a mixture of PBS or PBG, and 0.1% Triton X-100, respectively. The haemolytic activity was calculated as the percentage of total haemoglobin released compared with that released by incubation with 0.1% Triton X-100.

### Fruit infection assays

The inoculation of *P*. *digitatum* strains on non-treated freshly harvested orange fruits (*Citrus sinensis* L. Osbeck cv Navelina) was conducted as previously described^47^. Briefly, three replicates of five fruits were inoculated with 5 µl of conidial suspension (5 × 10^4^ conidia/ml) at four wounds around the equator. Orange fruits were stored at 20 °C and 90% relative humidity. Each wound was scored daily for infection symptoms on consecutive post inoculation days.

### Data availability

All data generated or analyzed during this study are included in this published article (and its Supplementary Information files).

## Electronic supplementary material

Supplementary Information
