# Supplementary material for: Efficient production and characterization of the novel and highly active antifungal protein AfpB from Penicillium digitatum
Source: Sci Rep. 2017 Nov 7;7:14663. doi: 10.1038/s41598-017-15277-w (PMC5677034; doi:10.1038/s41598-017-15277-w)
Supplement: Supplementary file 1 — Supplementary Information [file 41598_2017_15277_MOESM1_ESM.pdf]

Supplementary Information

Efficient production and functional characterization of the highly active antifungal protein AfpB from *Penicillium digitatum*

Sandra Garrigues[1], Mónica Gandía[1], Crina Popa[2], Attila Borics[3], Florentine Marx[4], María Coca[2], Jose F. Marcos[1], Paloma Manzanares[1]*

[1]Department of Biotechnology, Instituto de Agroquímica y Tecnología de Alimentos (IATA), Consejo Superior de Investigaciones Científicas (CSIC), Paterna, Valencia, Spain.

[2]Centre for Research in Agricultural Genomics (CRAG), CSIC-IRTA-UAB-UB. Edifici CRAG, Bellaterra, Barcelona, Spain

[3]Institute of Biochemistry, Biological Research Centre of Hungarian Academy of Sciences, Szeged, Hungary.

[4]Biocenter, Division of Molecular Biology, Medical University of Innsbruck, Innsbruck, Austria.

*Correspondence:

Paloma Manzanares

pmanz@iata.csic.es

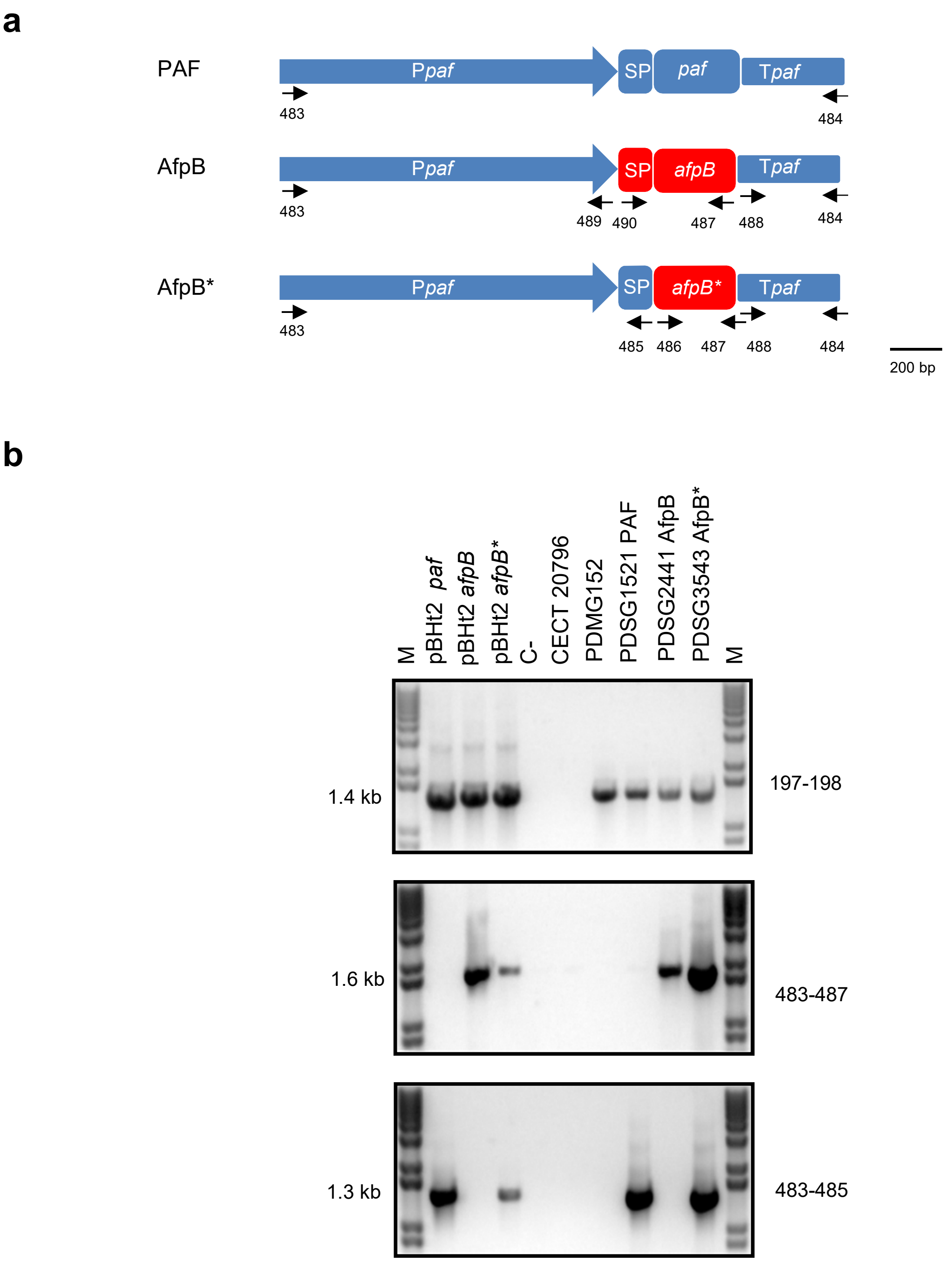

Supplementary Figure S1. Molecular characterization of *P. digitatum* AfpB and AfpB* producer strains. **a)** Representation of the expression systems used to obtain AfpB producer strains. The first diagram constitutes the schematic representation of the *P. chrysogenum*-based expression cassette (PAF); in blue: *paf* promoter (P*paf*), *paf* gene including the *paf* SP-pro sequence (*paf* SP), and *paf* terminator (T*paf*). The second diagram (AfpB) represents the genetic construction with the full-length AfpB coding sequence (in red) cloned under the control of the P*paf* and T*paf* from *P. chrysogenum*. The third part (AfpB*) corresponds to the genetic construction with the *in silico* predicted AfpB coding sequence (*afpB**) cloned under the control of the P*paf*, *paf* SP-pro sequence and T*paf*. All primers used for each construction generation and PCR analyses are located in the figure. **b**) PCR amplification of genomic DNA of the distinct *P. digitatum* strains with different primer pairs as indicated. Controls are the wild type strain CECT 20796, the *afpB*^{C} strain PDMG152, and the PAF producer strain PDSG1521. Additional controls were pBHt2 plasmids carrying the *paf, afpB* and *afpB** gene constructions and no DNA control (C-). The 1.4 kb bands (primers OJM197 and OJM198) correspond to the hygromycin resistant cassette (*hph*), which is present in all mutants and plasmid constructions. The 1.6 kb bands (primers OJM483 and OJM 487) correspond to the amplified region from the 5’ P*paf* to the 3’ *afpB* gene, only present in AfpB producer strains and *afpB* plasmid constructions. The 1.3 kb bands (OJM483-OJM485) correspond to the amplified region from 5’ P*paf* to the 3’ *paf* SP, only present in the PAF producer strain PDSG1521, AfpB* producer strain PDSG3543, and its corresponding plasmids.

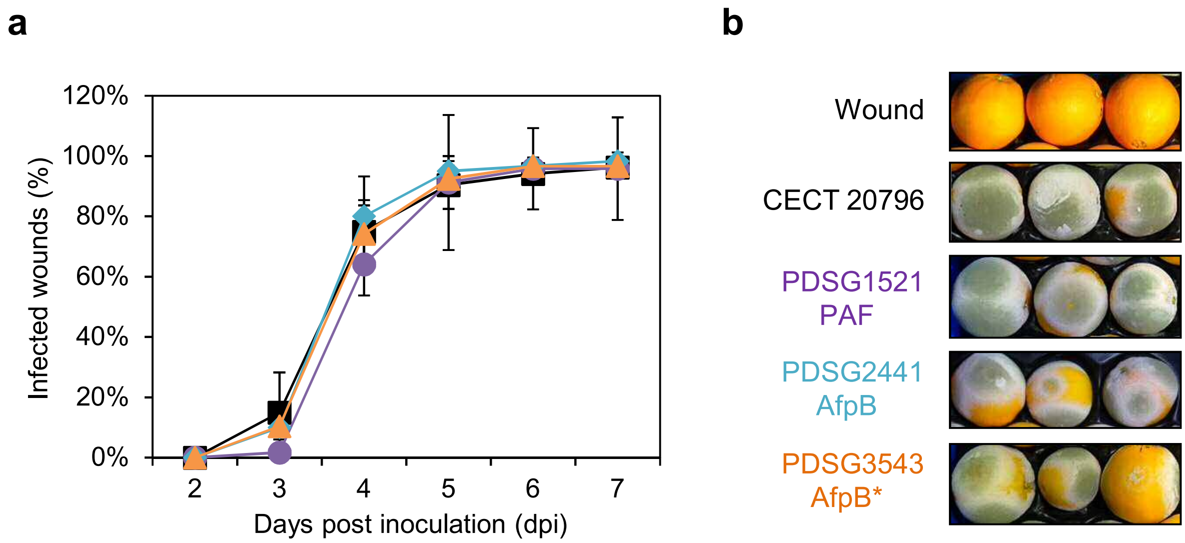

Supplementary Figure S2. Virulence assays of *P. digitatum* strains on orange fruits. a) Incidence of infection caused by the parental strain CECT 20796 (black squares), and transformants PDSG1521 (purple circles), PDSG2441 (blue diamonds), and PDSG3543 (orange triangles), which carry PAF, AfpB and AfpB* genetic constructions, respectively. Data are referred as mean values ± standard deviation of three replicates. **b**) Representative images of orange fruits infected by the indicated strains at 7 dpi.

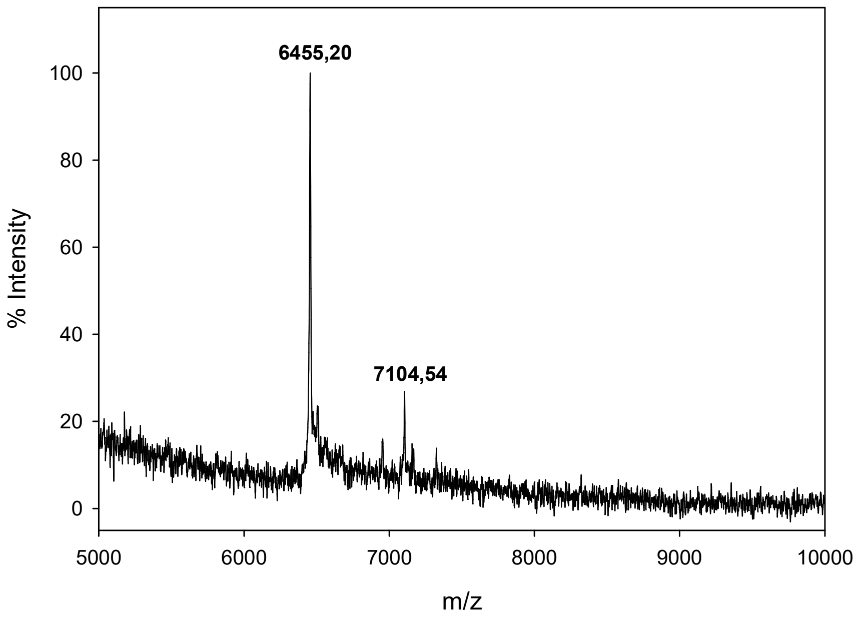

Supplementary Figure S3. MALDI-TOF MS data showing the isotopic average molecular mass (m/z) of AfpB* produced in the yeast *P. pastoris*. The predominant signal of 6455.20 Da corresponds to the calculated molecular mass of the oxidized protein AfpB* and the proper processing of the yeast α-factor signal peptide sequence (α-factor SS). The additional signal of 7104.54 Da can not be explained by any incorrect processing of the α-factor SS in *P. pastoris.*

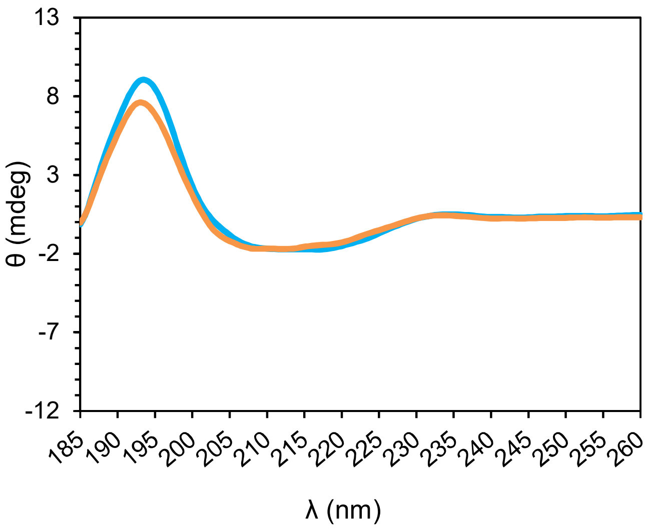

Supplementary Figure S4. ECD spectra of both *P. digitatum* AfpB variants at 25 ºC. Spectra obtained for AfpB (light blue) and AfpB* (orange) in H_2O at 25 ºC. Spectra show that both protein variants have practically the same folding at 25 ºC.

a

b

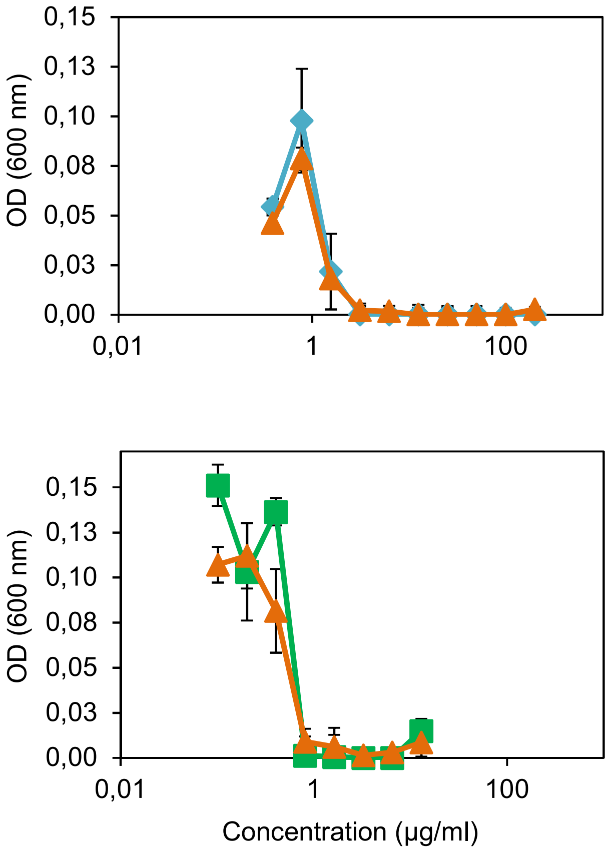

Supplementary Figure S5. Comparative study of the antifungal activity of AfpB and AfpB* variants . In this figure, two independent assays are shown. **a**) In vitro antifungal activity of AfpB (blue diamonds) and AfpB* (orange triangles) produced in *P. digitatum* against *P. digitatum* wild type strain after 72 h of growth at 25 ºC. **b**) Antifungal activity of AfpB* produced in *P. digitatum* (orange triangles) and PpAfpB* produced in *P. pastoris* (green squares) against *P. digitatum* wild type strain after 72 h of growth at 25 ºC. Data are referred as mean values ± s. d. of three replicates.

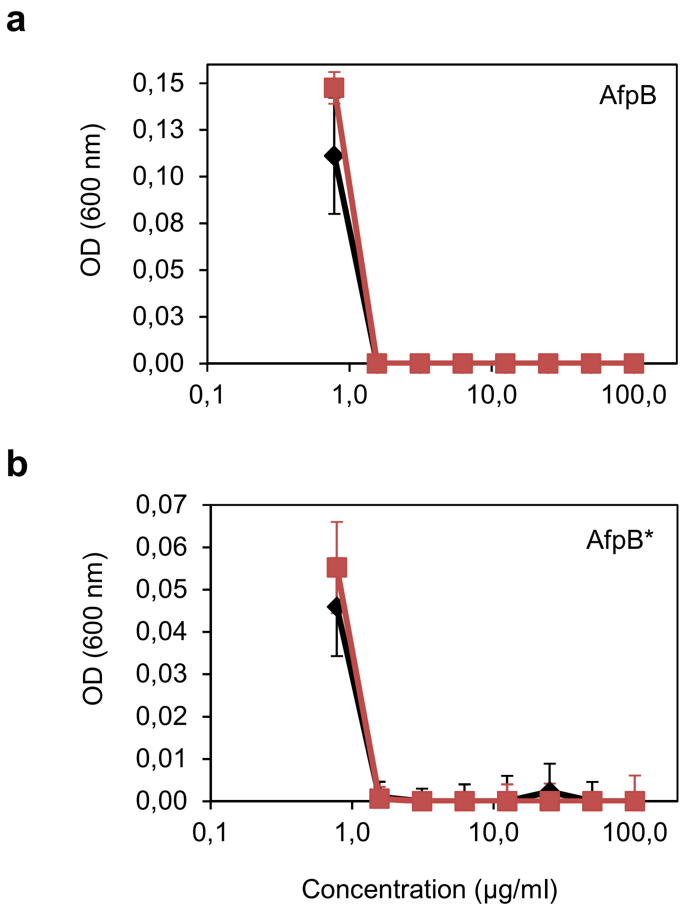

Supplementary Figure S6. Comparative study of the antifungal activity of both AfpB and AfpB* variants after treatment with proteinase K. **a**) Dose-response curve of *P. digitatum* CECT 20796 inhibition by AfpB (black diamonds) and AfpB after treatment with 100 µg/ml of proteinase K for 20 h (red squares). **b**) Dose-response curve of *P. digitatum* CECT 20796 inhibition by AfpB* (black diamonds) and AfpB* after treatment with 100 µg/ml of proteinase K for 20 h (red squares).

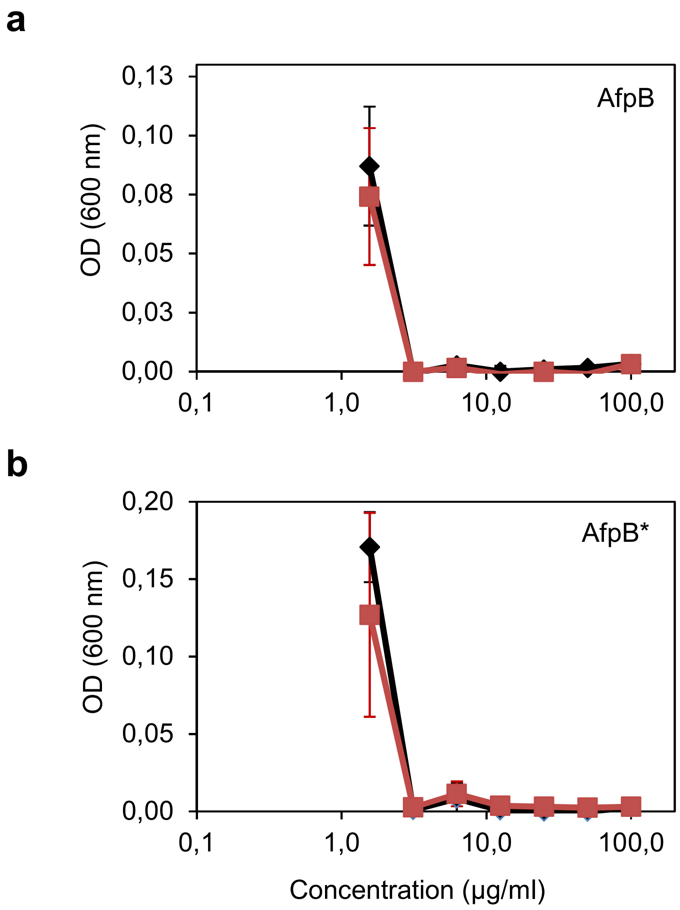

Supplementary Figure S7. Comparative study of the antifungal activity of both AfpB and AfpB* variants after heat treatment. **a**) Dose-response curve of *P. digitatum* CECT 20796 inhibition by AfpB (black diamonds) and AfpB after treatment at 95 ºC for 5 min (red squares). **b**) Dose-response curve of *P. digitatum* CECT 20796 inhibition by AfpB* (black diamonds) and AfpB* after treatment at 95 ºC for 5 min (red squares).

Supplementary Table S1. Primers used in this study.

Name	Use*	Sequence 5´- 3´**	Tm*** (ºC)	Restriction sites	Gene	Description
OJM483	F	AT**CCCGGG**GAATTCAGAGAGCTTTTCGTACG	60	*Xma*I	*paf* promoter	Fungal transformation and transformant verification
OJM484	R	AT**TCTAGA**GCAGCAGTTTGATAGTTATCCCT	60	*Xba*I	*paf* terminator	Fungal transformation
OJM485	R	CAGGACACCGGCCTCAGCCC	60		*paf* pre-pro-sequence	Fungal transformation and transformant verification
OJM486	F	GGCTGAGGCCGGTGTCCTGAGTAAATACGGAGGAGTAAGTT	60		*afpB**	Fungal transformation
OJM487	R	GGTGATCGCAGAGACCATTCAAACTGGAGTCTGGCAGTC	60		*afpB*	Fungal transformation and transformant verification
OJM488	F	ATGGTCTCTGCGATCACCAGG	60		*paf* terminator	Fungal transformation
OJM489	R	TATGAAGGGCTTGAGATGATGATC	60		*paf* promoter	Fungal transformation
OJM490	F	CATCATCTCAAGCCCTTCATAATGCAGATTACCAGCATTGCC	60		*afpB*	Fungal transformation
OJM467	R	AGTCAACCCTCCTGTGGTG	56		*afpB*	DNA Sequencing
OJM491	F	CCACTTTAACCTTCTCCAGA	56		*paf* promoter	DNA Sequencing
OJM380	F	GTAATACGACTCACTATAGGG	56		*sp6* promoter	DNA Sequencing
OJM381	R	CATTTAGGTGACACTATAGAATAC	56		*T7* promoter	DNA Sequencing
OJM197	F	C**GTTAAC**TGATATTGAAGGAGCAT	60	*Hpa*I	*hph*	Fungal transformant verification
OJM198	R	T**GTTAAC**TGGTTCCCGGTCGG	60	*Hpa*I	*hph*	Fungal transformant verification
MO3	F	G**CTCGAG**AAAAGAAGTAAATAC	56	*Xho*I	*Kex2* signal cleavage + *afpB**	Yeast transformation and transformant verification
MO4	R	CC**TCTAGA**TCAAACTGGAGTCTG	62	*Xba*I	*Kex2* signal cleavage + *afpB**	Yeast transformation and transformant verification

* F: forward; R: reverse.
** Restriction sites are highlighted in bold.
*** Tm: Temperature of annealing.
